# Top 100 cited classical articles in sentinel lymph nodes biopsy for breast cancer

**DOI:** 10.3389/fonc.2023.1170464

**Published:** 2023-10-09

**Authors:** Xinrui Liang, Yu Wang, Guanghua Fu, Pingmig Fan, Ke Ma, Xu-Chen Cao, Guang-Xun Lin, Wu-ping Zheng, Peng-fei Lyu

**Affiliations:** ^1^Breast Cancer Center, Chongqing Cancer Institute, Chongqing University Cancer Hospital, Chongqing, China; ^2^Department of Breast Surgery, The First Affiliated Hospital of Hainan Medical University, Haikou, China; ^3^The First Department of Breast Cancer, National Clinical Research Center for Cancer, Key Laboratory of Cancer Prevention and Therapy, Key Laboratory of Breast Cancer Prevention and Therapy, Ministry of Education, Tianjin’s Clinical Research Center for Cancer, Tianjin Medical University Cancer Institute and Hospital, Tianjin Medical University, Tianjin, China; ^4^Department of Thyroid and Breast Surgery, The Second Affiliated Hospital of Hainan Medical University, Haikou, China; ^5^Department of Orthopedics, The First Affiliated Hospital of Xiamen University, School of Medicine, Xiamen University, Xiamen, China

**Keywords:** breast cancer, sentinel lymph node, biopsy, trends, bibliometric

## Abstract

**Background:**

The sentinel lymph node biopsy (SLNB) takes on a critical significance in breast cancer surgery since it is the gold standard for assessing axillary lymph node (ALN) metastasis and determining whether to perform axillary lymph node dissection (ALND). A bibliometric analysis is beneficial to visualize characteristics and hotspots in the field of sentinel lymph nodes (SLNs), and it is conducive to summarizing the important themes in the field to provide more insights into SLNs and facilitate the management of SLNs.

**Materials and methods:**

Search terms relating to SLNs were aggregated and searched in the Web of Science core collection database to identify the top 100 most cited articles. Bibliometric tools were employed to identify and analyze publications for annual article volume, authors, countries, institutions, keywords, as well as hotspot topics.

**Results:**

The period was from 1998 to 2018. The total number of citations ranged from 160 to 1925. LANCET ONCOLOGY and JAMA-JOURNAL OF THE AMERICAN MEDICAL ASSOCIATION were the top two journals in which the above articles were published. Giuliano, AE was the author with the highest number of articles in this field with 15. EUROPEAN INST ONCOL is the institution with the highest number of publications, with 35 articles. Hotspots include the following 4 topics, false-negative SLNs after neoadjuvant chemotherapy; prediction of metastatic SLNs; quality of life and postoperative complications; and lymphography of SLNs.

**Conclusion:**

This study applies bibliometric tools to analyze the most influential literature, the top 100 cited articles in the field of SLNB, to provide researchers and physicians with research priorities and hotspots.

## 1 Introduction

Breast cancer has become the disease with the highest morbidity and mortality among women in most countries (1). The axillary lymph node (ALN) status is one of the most important indicators for evaluating the prognosis of breast cancer (2), and axillary lymph node dissection (ALND) has always been considered the gold standard for assessing the metastasis of ALNs, and it has been commonly employed in the surgical treatment of breast cancer. However, as a result of the obstruction of the axillary lymphatic system easily by axillary surgery, which can result in a series of complications (e.g., lymphedema of the affected limb, limited shoulder joint movement, and decreased muscle strength (3–5)), ALND has resulted in great inconvenience to the patients in postoperative life.

With the improvement of early diagnosis and comprehensive treatment of breast cancer, de-escalation has been more generally recommended as the surgical treatment of breast cancer. A considerable number of researches have confirmed that the sentinel lymph node (SLN) of breast cancer is the first-stop lymph node draining the primary tumor, as well as the first-stop lymph node draining the entire breast organ (6, 7). Since this concept has been progressively recognized, sentinel lymph node biopsy (SLNB) has been extensively employed in clinical trials. SLNB can accurately stage the ALN status of breast cancer (8–12) while effectively reducing the incidence of postoperative complications (13–17). It has replaced conventional axillary surgery and become the standard surgical treatment for early breast cancer (18, 19).

Bibliometric analysis is effective in investigating the development of a field and identifying vital research hotspots (20). It is imperative to use the bibliometric analysis that can provide various quantitative indicators of the number of publications, scientific achievements, and the effect of authors (21, 22) to identify the trends and hotspots of SLNB and then help scholars explore new directions for future researches in the academic realm.

## 2 Materials and methods

### 2.1 Data source

The publication on sentinel lymph nodes of breast cancer retrieved 7076 articles in the Web of Science core collection database (WoSCC) from 1998 to August 2022. The search strategy is presented as follows: “sentinel node*” or “sentinel lymph node*” or “sentinel lymphadenectomy” or “SLNs” AND “breast cancer” or “breast carcinoma” or “breast tumor*” or “breast neoplasm”. 3614 English articles were included. Lastly, the 100 most cited publications were collected. Two authors (Pengfei Lyu and Pingming Fan) assessed the retrieved data respectively to identify the literature relating to SLNB in breast cancer. If there are any opinions, another author (Ke Ma) will be consulted, and a consensus will be reached through discussion.

### 2.2 Statistical analysis

Data were input into the tools of bibliometrics. The analysis was conducted using RStudio (version 4.1.3), gCLUTO (version 1.0), and VOSviewer (version 1.6.14), which were adopted to build networks of bibliographic couplings based on keywords and co-occurrence analysis, among others for the visual analysis of the network.

## 3 Results

### 3.1 Main information

The period was from 1998 to 2018; the sources (journals) were 29; the average number of citations per document was 365.6; the average number of citations per document per year was 24.8. Article type: article 75; proceedings paper 18; editorial material 1; review 6.

The total number of citations ranged from 160 to 1925. The most cited articles have been written by Giuliano, AE, and so forth published in the JAMA-JOURNAL OF THE AMERICAN MEDICAL ASSOCIATION in 2011 on a comparative study of ALND and non-ALND in breast cancer with sentinel lymph node metastasis. The top 100 most cited articles in the field of SLNB for breast cancer are listed in **Table 1**. **Table 2** lists the annual average citation (citation rate) of the top 10 articles relating to SLNB. Articles published by Giuliano, AE, and so forth occupied four of them, and the period was from 1994 to 2017. LANCET ONCOLOGY and JAMA-JOURNAL OF THE AMERICAN MEDICAL ASSOCIATION were the top two journals of the above articles published.

**Table 1:** The top 100 most cited articles on SLNB.

Rank	First author	Article title	Journal	Publication Years	Total citation
1	Giuliano,,AE	Lymphatic mapping and sentinel lymphadenectomy for breast-cancer.	ANNALS OF SURGERY	1994	2,047
2	Giuliano, AE	Axillary Dissection vs No Axillary Dissection in Women With Invasive Breast Cancer and Sentinel Node Metastasis A Randomized Clinical Trial	JAMA-JOURNAL OF THE AMERICAN MEDICAL ASSOCIATION	2011	1,924
3	Krag, D	The sentinel node in breast cancer - A multicenter validation study	NEW ENGLAND JOURNAL OF MEDICINE	1998	1,536
4	Veronesi, U	A randomized comparison of sentinel-node biopsy with routine axillary dissection in breast cancer	NEW ENGLAND JOURNAL OF MEDICINE	2003	1,496
5	Mansel, RE	Randomized multicenter trial of sentinel node biopsy versus standard axillary treatment in operable breast cancer: The ALMANAC trial	JOURNAL OF THE NATIONAL CANCER INSTITUTE	2006	1,102
6	Krag, DN	Sentinel-lymph-node resection compared with conventional axillary-lymph-node dissection in clinically node-negative patients with breast cancer: overall survival findings from the NSABP B-32 randomised phase 3 trial	LANCET ONCOLOGY	2010	1,038
7	Donker, M	Radiotherapy or surgery of the axilla after a positive sentinel node in breast cancer (EORTC 10981-22023 AMAROS): a randomised, multicentre, open-label, phase 3 non-inferiority trial	LANCET ONCOLOGY	2014	623 892
8	Giuliano, AE	Locoregional Recurrence After Sentinel Lymph Node Dissection With or Without Axillary Dissection in Patients With Sentinel Lymph Node Metastases	ANNALS OF SURGERY	2010	859
9	Giuliano, AE	Sentinel lymphadenectomy in breast cancer	JOURNAL OF CLINICAL ONCOLOGY	1997	827
10	Boughey, JC	Sentinel Lymph Node Surgery After Neoadjuvant Chemotherapy in Patients With Node-Positive Breast Cancer The ACOSOG Z1071 (Alliance) Clinical Trial	JAMA-JOURNAL OF THE AMERICAN MEDICAL ASSOCIATION	2013	763
11	Galimberti, V	Axillary dissection versus no axillary dissection in patients with sentinel-node micrometastases (IBCSG 23-01): a phase 3 randomised controlled trial	LANCET ONCOLOGY	2013	756
12	Krag, DN	Technical outcomes of sentinel-lymph-node resection and conventional axillary-lymph-node dissection in patients with clinically node-negative breast cancer: results from the NSABP B-32 randomised phase III trial	LANCET ONCOLOGY	2007	721
13	Kuehn, T	Sentinel-lymph-node biopsy in patients with breast cancer before and after neoadjuvant chemotherapy (SENTINA): a prospective, multicentre cohort study	|LANCET ONCOLOGY	2013	711
14	GIULIANO, AE	Improved axillary staging of breast-cancer with sentinel lymphadenectomy	ANNALS OF SURGERY	1995	709
15	Van Zee, KJ	A nomogram for predicting the likelihood of additional nodal metastases in breast cancer patients with a positive sentinel node biopsy	ANNALS OF SURGICAL ONCOLOGY	2003	605
16	Giuliano, AE	Effect of Axillary Dissection vs No Axillary Dissection on 10-Year Overall Survival Among Women With Invasive Breast Cancer and Sentinel Node Metastasis The ACOSOG Z0011 (Alliance) Randomized Clinical Trial	JAMA-JOURNAL OF THE AMERICAN MEDICAL ASSOCIATION	2017	603
17	Veronesi, U	Sentinel lymph node biopsy and axillary dissection in breast cancer: Results in a large series	JNCI-JOURNAL OF THE NATIONAL CANCER INSTITUTE	1999	580
18	Lucci, A	Surgical complications associated with sentinel lymph node dissection (SLND) plus axillary lymph node dissection compared with SLND alone in the American College of Surgeons oncology Group trial Z0011	JOURNAL OF CLINICAL ONCOLOGY	2007	551
19	Borgstein, P	Sentinel lymph node biopsy in breast cancer: Guidelines and pitfalls of lymphoscintigraphy and gamma probe detection	JOURNAL OF THE AMERICAN COLLEGE OF SURGEONS	1998	551
20	Morton, DL	Sentinel node biopsy for early-stage melanoma - Accuracy and morbidity in MSLT-I, an international multicenter trial	ANNALS OF SURGERY	2005	546
21	Lyman, GH	Sentinel Lymph Node Biopsy for Patients With Early-Stage Breast Cancer: American Society of Clinical Oncology Clinical Practice Guideline Update	JOURNAL OF CLINICAL ONCOLOGY	2014	543
22	Troyan, SL	The FLARE((TM)) Intraoperative Near-Infrared Fluorescence Imaging System: A First-in-Human Clinical Trial in Breast Cancer Sentinel Lymph Node Mapping	ANNALS OF SURGICAL ONCOLOGY	2009	496
23	Cox, CE	Guidelines for sentinel node biopsy and lymphatic mapping of patients with breast cancer	ANNALS OF SURGERY	1998	487
24	Turner, RR	Histopathologic validation of the sentinel lymph node hypothesis for breast carcinoma	ANNALS OF SURGERY	1997	469
25	McMasters, KM	Sentinel lymph node biopsy for breast cancer: A suitable alternative to routine axillary dissection in multi-institutional practice when optimal technique is used	JOURNAL OF CLINICAL ONCOLOGY	2000	445
26	Giuliano, AE	Prospective observational study of sentinel lymphadenectomy without further axillary dissection in patients with sentinel node-negative breast cancer	JOURNAL OF CLINICAL ONCOLOGY	2000	430
27	McLaughlin, SA	Prevalence of Lymphedema in Women With Breast Cancer 5 Years After Sentinel Lymph Node Biopsy or Axillary Dissection: Objective Measurements	JOURNAL OF CLINICAL ONCOLOGY	2008	408
28	Rossi, EC	A comparison of sentinel lymph node biopsy to lymphadenectomy for endometrial cancer staging (FIRES trial): a multicentre, prospective, cohort study	LANCET ONCOLOGY	2017	405
29	Schrenk, P	Morbidity following sentinel lymph node biopsy versus axillary lymph node dissection for patients with breast carcinoma	CANCER	2000	403
30	Wilke, LG	Surgical complications associated with sentinel lymph node biopsy: Results from a prospective international cooperative group trial	ANNALS OF SURGICAL ONCOLOGY	2006	395
31	Boileau, JF	Sentinel Node Biopsy After Neoadjuvant Chemotherapy in Biopsy-Proven Node-Positive Breast Cancer: The SN FNAC Study	JOURNAL OF CLINICAL ONCOLOGY	2015	393
32	Purushotham, AD	Morbidity after sentinel lymph node biopsy in primary breast cancer: Results from a randomized controlled trial	JOURNAL OF CLINICAL ONCOLOGY	2005	373
33	Mamounas, EP	Sentinel node biopsy after neoadjuvant chemotherapy in breast cancer: Results from National Surgical Adjuvant Breast and Bowel Project Protocol B-27	JOURNAL OF CLINICAL ONCOLOGY	2005	362
34	Veronesi,U	Sentinel Lymph Node Biopsy in Breast Cancer Ten-Year Results of a Randomized Controlled Study	ANNALS OF SURGERY 251	2010	353
35	Fleissig, A	Post-operative arm morbidity and quality of life. Results of the ALMANAC randomised trial comparing sentinel node biopsy with standard axillary treatment in the management of patients with early breast cancer	BREAST CANCER RESEARCH AND TREATMENT	2006	346
36	O'Hea, BJ	Sentinel lymph node biopsy in breast cancer: Initial experience at Memorial Sloan-Kettering Cancer Center	JOURNAL OF THE AMERICAN COLLEGE OF SURGEONS	1998	341
37	Veronesi, U	Sentinel-lymph-node biopsy as a staging procedure in breast cancer: update of a randomised controlled study	LANCET ONCOLOGY	2006	331
38	Chu, KU	Do all patients with sentinel node metastasis from breast carcinoma need complete axillary node dissection?	ANNALS OF SURGERY	1999	328
39	Song, KH	Near-Infrared Gold Nanocages as a New Class of Tracers for Photoacoustic Sentinel Lymph Node Mapping on a Rat Model	NANO LETTERS	2009	309
40	Naik, AM	The risk of axillary relapse after sentinel lymph node biopsy for breast cancer is comparable with that of axillary lymph node dissection - A follow-up study of 4008 procedures	ANNALS OF SURGERY	2004	302
41	Ashikaga, T	Morbidity Results From the NSABP B-32 Trial Comparing Sentinel Lymph Node Dissection Versus Axillary Dissection	|JOURNAL OF SURGICAL ONCOLOGY	2010	292
42	Xing, Y	Meta-analysis of sentinel lymph node biopsy after preoperative chemotherapy in patients with breast cancer	BRITISH JOURNAL OF SURGERY	2006	288
43	Tafra, L	Multicenter trial of sentinel node biopsy for breast cancer using both technetium sulfur colloid and isosulfan blue dye	ANNALS OF SURGERY	2001	281
44	Giuliano, AE	Locoregional Recurrence After Sentinel Lymph Node Dissection With or Without Axillary Dissection in Patients With Sentinel Lymph Node Metastases Long-term Follow-up From the American College of Surgeons Oncology Group (Alliance) ACOSOG Z0011 Randomized Trial	ANNALS OF SURGERY	2016	274
45	Gentilini, O and Veronesi, U	Abandoning sentinel lymph node biopsy in early breast cancer? A new trial in progress at the European Institute of Oncology of Milan (SOUND: Sentinel node vs Observation after axillary UltraSouND)	BREAST	2012	259
46	Bilimoria, KY	Comparison of Sentinel Lymph Node Biopsy Alone and Completion Axillary Lymph Node Dissection for Node-Positive Breast Cancer	|JOURNAL OF CLINICAL ONCOLOGY	2009	259
47	Reynolds	Sentinel lymph node biopsy with metastasis: Can axillary dissection be avoided in some patients with breast cancer?	JOURNAL OF CLINICAL ONCOLOGY	1999	253
48	Langer, I	Morbidity of sentinel lymph node biopsy (SLN) alone versus SLN and completion axillary lymph node dissection after breast cancer surgery - A prospective Swiss 5multicenter study on 659 patients	ANNALS OF SURGERY	2007	241
49	Turner, RR	Pathologic features associated with nonsentinel lymph node metastases in patients with metastatic breast carcinoma in a sentinel lymph node	CANCER	2000	240
50	Zavagno, G	A randomized clinical trial on sentinel lymph node biopsy versus axillary lymph node dissection in breast cancer - Results of the sentinella/GIVOM trial	ANNALS OF SURGERY	2008	239
51	Goyal, A	Factors affecting failed localisation and false-negative rates of sentinel node biopsy in breast cancer - results of the ALMANAC validation phase	BREAST CANCER RESEARCH AND TREATMENT	2006	235
52	Boughey, JC	Identification and Resection of Clipped Node Decreases the False-negative Rate of Sentinel Lymph Node Surgery in Patients Presenting With Node-positive Breast Cancer (T0-T4, N1-N2) Who Receive Neoadjuvant Chemotherapy: Results From ACOSOG Z1071 (Alliance)	ANNALS OF SURGERY	2016	233
53	Klauber-DeMore, N	Sentinel lymph node biopsy: Is it indicated in patients with high-risk ductal carcinoma-in-situ and ductal carcinoma-in-situ with microinvasion?	ANNALS OF SURGICAL ONCOLOGY	2000	229
54	Deurloo, EE	Reduction in the number of sentinel lymph node procedures by preoperative ultrasonography of the axilla in breast cancer	EUROPEAN JOURNAL OF CANCER	2003	226
55	Klimberg, VS	Subareolar versus peritumoral injection for location of the sentinel lymph node	ANNALS OF SURGERY	1999	220
56	Cserni, G	Meta-analysis of non-sentinel node metastases associated with micrometastatic sentinel nodes in breast cancer	|BRITISH JOURNAL OF SURGERY	2007	216
57	Qian, CN	Preparing the "soil": The primary tumor induces vasculature reorganization in the sentinel lymph node before the arrival of metastatic cancer cells	CANCER RESEARCH	2006	215
58	Viale, G	Intraoperative examination of axillary sentinel lymph nodes in breast carcinoma patients	CANCER	1999	215
59	Yen, TWF	Predictors of invasive breast cancer in patients with an initial diagnosis of ductal carcinoma in situ: A guide to selective use of sentinel lymph node biopsy in management of ductal carcinoma in situ	JOURNAL OF THE AMERICAN COLLEGE OF SURGEONS	2005	212
60	Viale, G	Predicting the risk for additional axillary metastases in patients with breast carcinoma and positive sentinel lymph node biopsy	ANNALS OF SURGERY	2005	212
61	Hunt, KK	Sentinel Lymph Node Surgery After Neoadjuvant Chemotherapy is Accurate and Reduces the Need for Axillary Dissection in Breast Cancer Patients	ANNALS OF SURGERY	2009	206
62	Bevilacqua, JLB	Doctor, what are my chances of having a positive sentinel node? A validated nomogram for risk estimation	JOURNAL OF CLINICAL ONCOLOGY	2007	204
63	Barranger, E	An axilla scoring system to predict non-sentinel lymph node status in breast cancer patients with sentinel lymph node involvemen	BREAST CANCER RESEARCH AND TREATMENT	2005	204
64	Lyman, GH	Sentinel Lymph Node Biopsy for Patients With Early-Stage Breast Cancer: American Society of Clinical Oncology Clinical Practice Guideline Update	JOURNAL OF CLINICAL ONCOLOGY	2017	202
65	Nason, KS	Increased false negative sentinel node biopsy rates after preoperative chemotherapy for invasive breast carcinoma	CANCER	2000	200
66	Blanchard, DK	Relapse and morbidity in patients undergoing sentinel lymph node biopsy alone or with axillary dissection for breast cancer	ARCHIVES OF SURGERY	2003	199
67	Galimberti, V	Axillary dissection versus no axillary dissection in patients with breast cancer and sentinel-node micrometastases (IBCSG 23-01): 10-year follow-up of a randomised, controlled, phase 3 trial	LANCET ONCOLOGY	2018	195
68	van Diest, PJ	Reliability of intraoperative frozen section and imprint cytological investigation of sentinel lymph nodes in breast cancer	HISTOPATHOLOGY	1999	193
69	Kohrt, HE	New models and online calculator for predicting non-sentinel lymph node status in sentinel lymph node positive breast cancer patients	BMC CANCER	2008	192
70	McMasters, KM	Dermal injection of radioactive colloid is superior to peritumoral injection for breast cancer sentinel lymph node biopsy: Results of a multiinstitutional study	ANNALS OF SURGERY	2001	192
71	Breslin, TM	Sentinel lymph node biopsy is accurate after neoadjuvant chemotherapy for breast cancer	JOURNAL OF CLINICAL ONCOLOGY	2000	192
72	Erpelding, TN	Sentinel Lymph Nodes in the Rat: Noninvasive Photoacoustic and US Imaging with a Clinical US System	RADIOLOGY	2010	190
73	Schijven, MP	Comparison of morbidity between axillary lymph node dissection and sentinel node biopsy	EUROPEAN JOURNAL OF SURGICAL ONCOLOGY	2003	190
74	Kim, C	Sentinel Lymph Nodes and Lymphatic Vessels: Noninvasive Dual-Modality in Vivo Mapping by Using Indocyanine Green in Rats-Volumetric Spectroscopic Photoacoustic Imaging and Planar Fluorescence Imaging	RADIOLOGY	2010	188
75	Swenson, KK	Comparison of side effects between sentinel lymph node and axillary lymph node dissection for breast cancer	ANNALS OF SURGICAL ONCOLOGY	2002	187
76	Burak, WE	Sentinel lymph node biopsy results in less postoperative morbidity compared with axillary lymph node dissection for breast cancer	AMERICAN JOURNAL OF SURGERY	2002	186
77	Linehan, DC	Intradermal radiocolloid and intraparenchymal blue dye injection optimize sentinel node identification in breast cancer patients	ANNALS OF SURGICAL ONCOLOGY	1999	185
78	Giuliano, AE	Association of Occult Metastases in Sentinel Lymph Nodes and Bone Marrow With Survival Among Women With Early-Stage Invasive Breast Cancer	JAMA-JOURNAL OF THE AMERICAN MEDICAL ASSOCIATION	2001	180
79	Pal, A	A model for predicting non-sentinel lymph node metastatic disease when the sentinel lymph node is positive	BRITISH JOURNAL OF SURGERY	2008	180
80	Borgstein, PJ	Functional lymphatic anatomy for sentinel node biopsy in breast cancer - Echoes from the past and the periareolar blue method	ANNALS OF SURGERY	2000	180
81	Tagaya, N	Intraoperative identification of sentinel lymph nodes by near-infrared fluorescence imaging in patients with breast cancer	AMERICAN JOURNAL OF SURGERY	2008	179
82	Cody, HS	Complementarity of blue dye and isotope in sentinel node localization for breast cancer: Univariate and multivariate analysis of 966 procedures	ANNALS OF SURGICAL ONCOLOGY	2001	177
83	Montgomery, LL	Isosulfan blue dye reactions during sentinel lymph node mapping for breast cancer	ANESTHESIA AND ANALGESIA	2002	176
84	Ahmed, M	Novel techniques for sentinel lymph node biopsy in breast cancer: a systematic review	LANCET ONCOLOGY	2014	175
85	Viale, G	Histologic detection and clinical implications of micrometastases in axillary sentinel lymph nodes for patients with breast carcinoma	CANCER	2001	175
86	Coutant, C	Comparison of Models to Predict Nonsentinel Lymph Node Status in Breast Cancer Patients With Metastatic Sentinel Lymph Nodes: A Prospective Multicenter Study	JOURNAL OF CLINICAL ONCOLOGY	2009	174
87	Stearns, V	Sentinel lymphadenectomy after neoadjuvant chemotherapy for breast cancer may reliably represent the axilla except for inflammatory breast cancer	ANNALS OF SURGICAL ONCOLOGY	2002	174
88	Coutant,C	Comparison of Models to Predict Nonsentinel Lymph Node Status in Breast Cancer Patients With Metastatic Sentinel Lymph Nodes: A Prospective Multicenter Study	JOURNAL OF CLINICAL ONCOLOGY	2009	174
89	Kim, CH	Pathologic Ultrastaging Improves Micrometastasis Detection in Sentinel Lymph Nodes During Endometrial Cancer Staging	INTERNATIONAL JOURNAL OF GYNECOLOGICAL CANCER	2013	172
90	Straver, ME	Sentinel Node Identification Rate and Nodal Involvement in the EORTC 10981-22023 AMAROS Trial	ANNALS OF SURGICAL ONCOLOGY	2010	172
91	Morrow, M	Learning sentinel node biopsy: Results of a prospective randomized trial of two techniques	SURGERY	1999	170
92	Classe, JM	Sentinel Lymph Node Biopsy After Neoadjuvant Chemotherapy for Advanced Breast Cancer: Results of Ganglion Sentinelle et Chimiotherapie Neoadjuvante, a French Prospective Multicentric Study	JOURNAL OF CLINICAL ONCOLOGY	2009	169
93	Gill, G	Sentinel-Lymph-Node-Based Management or Routine Axillary Clearance? One-Year Outcomes of Sentinel Node Biopsy Versus Axillary Clearance (SNAC): A Randomized Controlled Surgical Trial	ANNALS OF SURGICAL ONCOLOGY	2009	166
94	Cserni, G	Pathological work-up of sentinel lymph nodes in breast cancer. Review of current data to be considered for the formulation of guidelines	EUROPEAN JOURNAL OF CANCER	2003	166
95	Miltenburg, DM	Meta-analysis of sentinel lymph node biopsy in breast cancer	JOURNAL OF SURGICAL RESEARCH	1999	165
96	Peintinger, F	Comparison of quality of life and arm complaints after axillary lymph node dissection vs sentinel lymph node biopsy in breast cancer patients	BRITISH JOURNAL OF CANCER	2003	164
97	Hirche, C	ICG fluorescence-guided sentinel node biopsy for axillary nodal staging in breast cancer	BREAST CANCER RESEARCH AND TREATMENT	2010	163
98	van Rijk, MC	Ultrasonography and fine-needle aspiration cytology can spare breast cancer patients unnecessary sentinel lymph node biopsy	ANNALS OF SURGICAL ONCOLOGY	2006	163
99	Bilchik, AJ	Universal application of intraoperative lymphatic mapping and sentinel lymphadenectomy in solid neoplasms	CANCER JOURNAL FROM SCIENTIFIC AMERICAN	1998	162
100	Sola, M	Complete Axillary Lymph Node Dissection Versus Clinical Follow-up in Breast Cancer Patients with Sentinel Node Micrometastasis: Final Results from the Multicenter Clinical Trial AATRM 048/13/2000	ANNALS OF SURGICAL ONCOLOGY	2013	161

**Table 2:** Annual average citation (citation rate) of top ten articles related to SLNB.

Rank	Citation rate	Original rank	First Author	Title	Journal	Country	Date
1	160.33	2	Giuliano, AE	Axillary Dissection vs No Axillary Dissection in Women with Invasive Breast Cancer and Sentinel Node Metastasis a Randomized Clinical Trial	JAMA-JOURNAL OF THE AMERICAN MEDICAL ASSOCIATION	USA	2011
2	100.5	16	Giuliano, AE	Effect of Axillary Dissection vs No Axillary Dissection on 10-Year Overall Survival Among Women with Invasive Breast Cancer and Sentinel Node Metastasis the ACOSOG Z0011 (Alliance) Randomized Clinical Trial	JAMA-JOURNAL OF THE AMERICAN MEDICAL ASSOCIATION	USA	2017
3	99.11	7	Donker, M;	Radiotherapy or surgery of the axilla after a positive sentinel node in breast cancer (EORTC 10981-22023 AMAROS): a randomised, multicentre, open-label, phase 3 non-inferiority trial	LANCET ONCOLOGY	Netherlands	2014
4	79.85	6	Krag, DN	Sentinel-lymph-node resection compared with conventional axillary-lymph-node dissection in clinically node-negative patients with breast cancer: overall survival findings from the NSABP B-32 randomised phase 3 trial	LANCET ONCOLOGY	USA	2010
5	76.3	10	Boughey, JC	Sentinel Lymph Node Surgery After Neoadjuvant Chemotherapy in Patients with Node-Positive Breast Cancer the ACOSOG Z1071 (Alliance) Clinical Trial	JAMA-JOURNAL OF THE AMERICAN MEDICAL ASSOCIATION	USA	2013
6	75.6	11	Galimberti, V;	Axillary dissection versus no axillary dissection in patients with sentinel-node micrometastases (IBCSG 23-01): a phase 3 randomised controlled trial	LANCET ONCOLOGY	Italy	2013
7	74.8	4	Veronesi, U	A randomized comparison of sentinel-node biopsy with routine axillary dissection in breast cancer	NEW ENGLAND JOURNAL OF MEDICINE	Italy	2003
8	71.1	13	Kuehn, T	Sentinel-lymph-node biopsy in patients with breast cancer before and after neoadjuvant chemotherapy (SENTINA): a prospective, multicentre cohort study	LANCET ONCOLOGY	Germany	2013
9	70.59	1	Giuliano,AE	Lymphatic Mapping and Sentinel Lymphadenectomy for Breast-Cancer	ANNALS OF SURGERY	USA	1994
10	66.46	29	Giuliano, AE	Locoregional Recurrence After Sentinel Lymph Node Dissection With or Without Axillary Dissection in Patients With Sentinel Lymph Node Metastases	ANNALS OF SURGERY	USA	2010

### 3.2 Analysis of countries, authors, journals, and institutions

**Figure 1:**
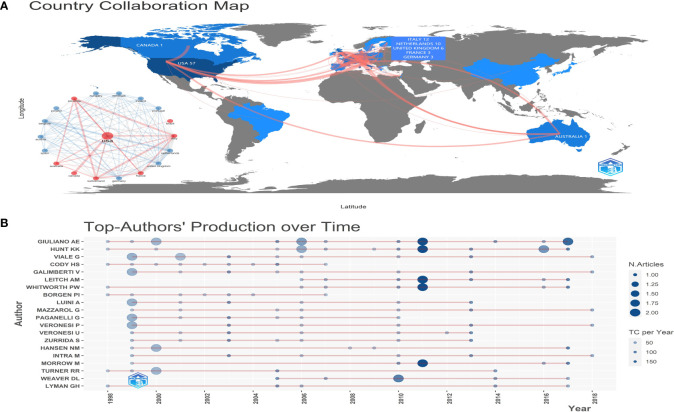
The map of annual scientific production and annual average article citations. **(A)** The annual scientific output of the top 100 most cited articles, **(B)** The average yearly number of article citations.

**Figure 1** shows annual scientific production and annual average article citations. The scientific output of the top 100 most cited articles reached the top in 1999(n=10, **Figure 1A**). The average yearly number of article citations reached 80.8 in 2017(**Figure 1B**).

**Figure 2:**
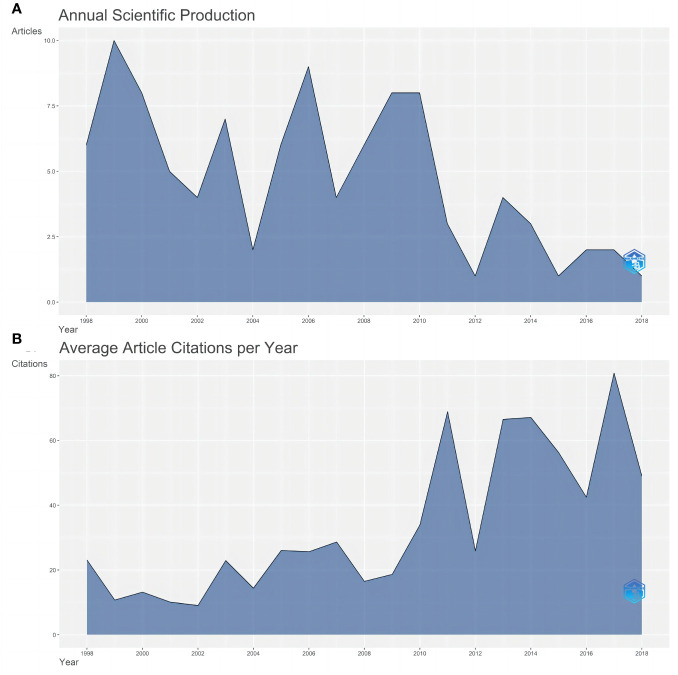
**(A)** Country Cooperation Map. The color depth represents the number of documents sent. The red line represents cooperation between countries. The thicker the line, the more the connections will be. **(B)** The top 20 authors’ production over time. The light blue circle represents the total citation (TC) per year, and the dark blue represents the number of articles.

57 of the 100 articles originated from the US, accounting for 57%. ITALY is the second-largest country with 12 pieces, far less than the US. **Figure 2A** illustrates the cooperation network of countries. As depicted in the figure, there are more than half of the articles from the US, as well as the most relationships with the US. Thus, the distribution of highly cited articles centered on the US has taken shape.

For the author, Giuliano AE has published 15 articles, followed by Hunt KK published 14 articles and Viale G published 10 articles. The top 20 authors’ production over time is shown in **Figure 2B**. High-yielding authors such as Giuliano AE and Hunt KK are also highly cited.

The most relevant sources (Top 20 journals) are presented in **Supplementary Figure 1**. To be specific, the JOURNAL OF CLINICAL ONCOLOGY and ANNALS OF SURGERY both published 16 articles, followed by the Journal of ANNALS OF SURGICAL ONCOLOGY published 13 articles.

The top 20 publishing institutions are displayed in **Supplementary Figure 2**. Since EUROPEAN INST ONCOL published the largest number of 35 articles, it was the institution that contributed the most to the research of the sentinel lymph nodes. MEM SLOAN KETTERING CANC CTR and UNIV TEXAS published 28 and 15 articles, respectively. The three-domain diagram of authors, institutions, and countries is depicted in **Supplementary Figure 3**. Most of the highly prolific authors and institutions are from the US and Italy.

### 3.3 Analysis of keywords, thematic terms, and hotspots clusters

**Figure 3:**
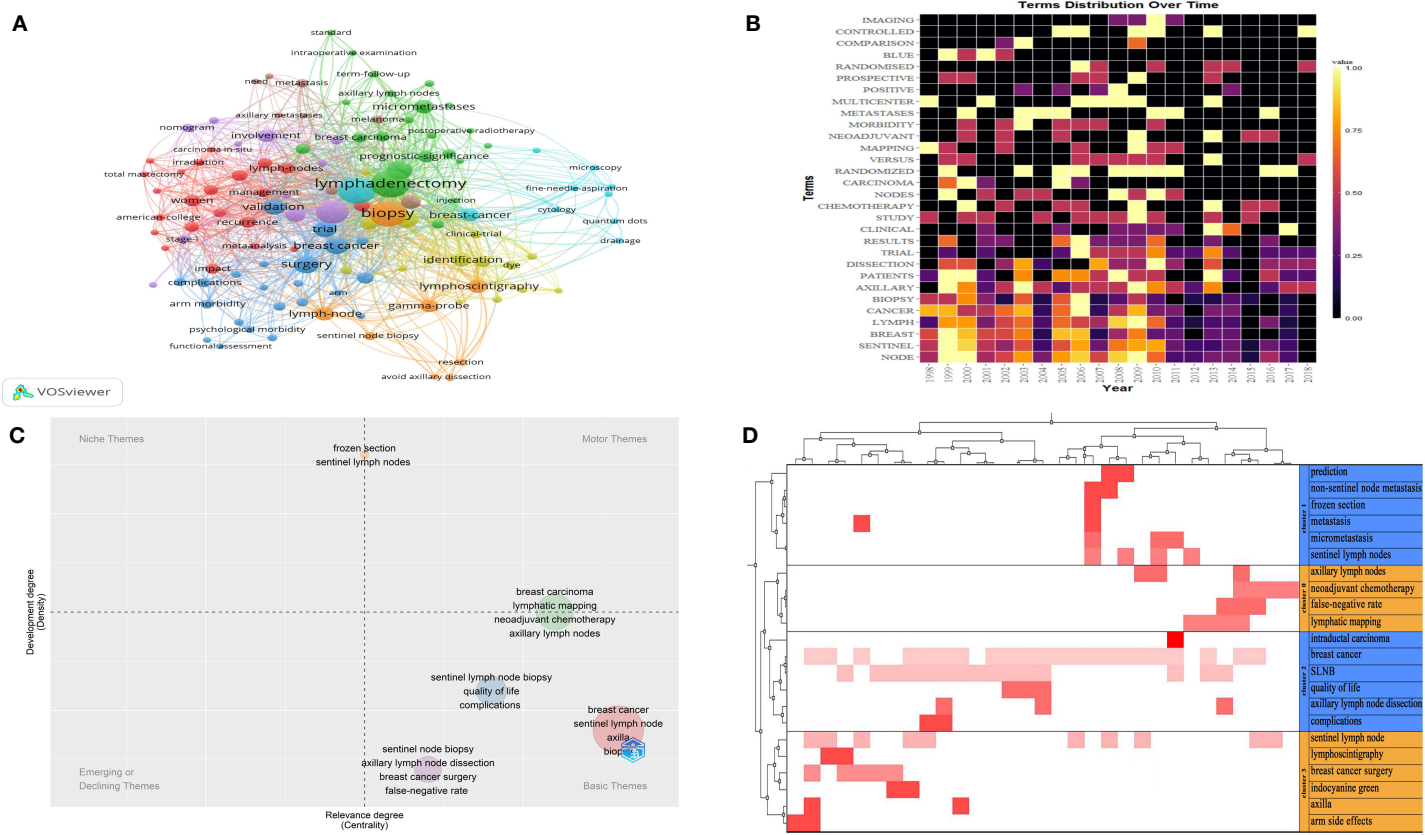
**(A)** The co-occurrence analysis profile of the keywords. The size of the node represents the frequency of occurrence. The same color represents the same theme. Connecting lines represent co-occurrence. **(B)** The distribution of high-frequency subject terms over time. Lighter colors represent higher frequency of occurrence, darker colors represent lower frequency of occurrence, and black colors represent a frequency of 0. **(C)** A thematic map of the SLNB. The horizontal coordinate represents the degree of relevance and the vertical coordinate represents the degree of development. **(D)** A heat map of the visualization of four clusters. The red color represents values in the original data, the dark color represents larger corresponding values and the white color represents corresponding values of 0.

**Figure 3A** presents the co-occurrence analysis profile of the keywords. The top ten frequency keywords are presented as follows: lymphadenectomy, biopsy, carcinoma, axillary dissection, surgery, breast cancer, validation trial, metastases or micro-metastases, multicenter. Hence, the above high-frequency keywords suggest that the research focus of the above 100 articles was on biopsies relating to sentinel lymph nodes, axillary lymph node dissection, multicenter clinical validation trials, and management of metastases or micro-metastases in axillary lymph nodes.

To avoid the bias of keywords representing the topic of the article, I have researched the subject terms (title keywords and abstract keywords). **Figure 3B** presents the distribution of high-frequency subject terms over time. The main subject terms were distributed year by year. We found that the main subject words occurred with high frequency from 1998 to 2013.

A thematic map of the SLNs is shown in **Figure 3C**. Basic themes are located in the lower right quadrant, including quality of life and complications after surgery; false-negative sentinel lymph node dissection; the impact of neoadjuvant chemotherapy on lymphatic imaging; and prediction of positive sentinel lymph nodes. The above small clustered themes are well-centered and poorly developed, suggesting that the above issues are extensively studied in the above 100 highly cited articles.

After the extraction of the subject terms and cluster analysis from 3 to 10, the result indicated that four clusters worked best, having the greatest in-group similarity and the smallest out-group similarity. The parameters related to four clusters are shown in the **Supplementary Table 1**.* A* heat map of the visualization of clusters is shown in **Figure 3D**. Using the semantics of the articles corresponding to the keywords we identified four major hotspots in SLNB research.

Cluster 0: False-negative sentinel lymph nodes after neoadjuvant chemotherapy

Cluster 1: Prediction of metastatic sentinel lymph nodes

Cluster 2: Quality of life and postoperative complications in sentinel lymph node biopsy versus axillary lymph node dissection

Cluster 3: Lymphography of the sentinel lymph nodes

## 4 Discussion

With a series of large samples of prospective clinical research on SLNB over the past few years, this operation has more crucial means for patients to assess lymph nodes status, staging, treatment plan formulation, and prognosis judgment of breast cancer and represents the development level of breast surgery to a certain extent (23, 24). There are considerable articles on sentinel lymph nodes from 1998 to 2022. In the above thousands of documents, many researchers have made outstanding contributions to the treatment of breast cancer, solved many clinical problems, and benefited most patients with breast cancer. In this study, bibliometrics was adopted to summarize the 100 most cited articles relating to SLNB. Moreover, characteristics, current hotspots, and possible trends can be assessed through the study of authors, countries, institutions, magazines, keywords, and so forth. Although most articles still use total citations to sort articles, there is a certain deviation in the early cited articles because they are time-dependent (25–27). Some literature is published in the recent period, whereas the number of citations is not enough to reflect the importance, and the articles with high citation rates are always worth exploring. Accordingly, we also analyzed the citation rate of articles (the average citations per year).

There is frequent cooperation between the US and European countries. The US not only accounts for a large proportion of the top 100 most cited articles. Most of the authors and institutions with great achievement come from the US, indicating that the US has made significant contributions to the research relating to SLNB in breast cancer and significantly affects the whole world. In terms of the research on reasons, the US government has given great financial support to sentinel lymph node researchers. The top 10 funding agencies are listed in **Supplementary Table 2**. Analysis of the keywords in the 100 most cited articles resulted in four major clusters, as follows.

***Cluster 0: False-negative sentinel lymph nodes after neoadjuvant chemotherapy***

Neoadjuvant chemotherapy (NAC) has been increasingly applied to locally advanced or early breast cancer to achieve the goal of radical or breast-conserving surgery at the lower stage. For patients without clinical metastasis of ALNs after NAC, the detection rate of SLNs accounted for 96%, the accuracy rate reached 99%, and the false negative rate (FNR) was 6%, similar to the FNR of SLNB in patients with early breast cancer who were not eligible for NAC (28). In other words, SLNB is adequately safe and feasible after NAC in this part of patients. For patients with clinical metastasis of ALNs, NAC may cause lymphatic vascular obstruction or fibrosis that changes the lymphatic drainage pathway, thus increasing the FNR of SLNB and affecting the judgment of tumor staging (29). Accordingly, ALND continues to be the standard treatment of patients whose metastatic ALNs disappeared by palpation and imaging assessment after NAC in routine clinical practice. Following the clinical guidelines, the FNR of SLNB should be lower than 10% (30), and on the premise of meeting this requirement, false-negative events may not affect the prognosis of patients after surgery (31). A meta-analysis enrolling 1521 patients who underwent SLNB or ALND after NAC in 23 articles showed that the accuracy rate of SLNB in evaluating the status of ALNs status was 89%, and the FNR was 13% which was higher than the threshold of 10% (32). As revealed by the results of relevant clinical trials, the FNR can be reduced to a certain extent by placing marker clips in lymph nodes before NAC, using ultrasound and other auxiliary examinations to assist in assessment before the operation, using the dye and nuclide dual tracer technology to map SLNs and increasing the number of SLNs during operation, as well as using immunohistochemical detection and defining the concept of SLNs strictly (33–38). However, the number of SLNs detected cannot be accurately predicted in clinical practice, the application of nuclide is limited, and even the clips placed by some patients may not be identified during the operation and others. As a result, other technologies (e.g., the application of targeted axillary lymph node dissection combining SLNB with marked lymph node biopsy (39)) and novel tracers (e.g., superparamagnetic iron oxide (40) and carbon nanoparticle suspension (41)) should be developed to provide the possibility for the safe use of SLNB in patients with clinically positive ALNs turning into negativity after NAC.

***Cluster 1: Prediction of metastatic sentinel lymph nodes***

Due to a considerable number of factors for the FNR of SLNB (42), many scholars have also tried to establish models for predicting metastatic SLNs, to select patients who are necessary to perform ALND after SLNB. The bibliometric analysis indicates that the nomogram is the main form of model construction, and the analysis of the above articles indicates that the size of the primary tumor, the number of SLN macrometastases and positive SLN resections, and peripheral vascular invasion are critical factors for additional metastasis of ALNs (43–49). The role of SLNB at the time that the risk of invasive disease on final pathology in patients with an initial diagnosis of ductal carcinoma *in situ* (ductal carcinoma in situ, DCIS) was sufficiently high and has not been well defined. The result of the study in the bibliometric analysis indicated that 55 years of age or younger, diagnosed by needle core biopsy, mammography with a size of at least 4 cm and high-grade DCIS were more likely to develop into invasive cancer. The presence of comedonecrosis and larger tumor sizes were the independent predictors of patients receiving SLNB, whereas the accessibility of the tumor was the only independent predictor of positive SLNs (50). Therefore, SLNB should not be performed routinely in all patients initially diagnosed with DCIS.

***Cluster 2: Quality of life and postoperative complications in sentinel lymph node biopsy versus axillary lymph node dissection***

ALND is the most accurate method to assess the status of ALNs in breast cancer, whereas it is also the main cause of postoperative complications (e.g., edema of the upper limb, pain, sensory and motor dysfunction). Since the screening methods for breast cancer are progressively enriched, the detection rate of early breast cancer increases year by year, and the proportion of new cases of breast cancer without the metastasis of ALNs also rises. If ALND is performed on all patients, most patients are excessive diagnosis and treatment, thus significantly affecting their quality of life and causing greater psychological stress.

Among the top 100 most cited articles, numerous articles have compared the complications and the quality of life of SLNB and ALND. The most cited and representative clinical trial called the ALMANAC trial showed that the postoperative situation of patients performing SLNB was superior to that of patients performing ALND in lymphedema, sensory disturbance, wound drainage, length of hospital stays, upper limb functional index, postoperative motor function recovery and mental illness (51, 52). A recent meta-analysis including 67 articles showed that SLNB was significantly lower than ALND in the prevalence of lymphedema and pain, and was also better than ALND in the range and strength of the affected limbs (53). In addition, a small-scale prospective clinical trial in the bibliometric statistics only reported the complications of SLNB which could not also be ignored. The article highlighted that older age and more SLNs resection are capable of increasing the incidence rate of axillary seroma, whereas this cannot affect the choice of axillary surgery for elderly patients with breast cancer and increase the number of SLNs dissection to reduce the false negative rate for surgeons (54).

Complications (e.g., lymphedema) can be currently treated by further surgery and should be consistent with the principle of prevention first and treatment as a supplement due to the difficulty of implementation and relatively high cost and long duration (55, 56). In brief, clinicians have sufficient evidence to consider that SLNB should be employed as the optimal operation for breast cancer patients with clinically negative ALNs.

***Cluster 3: Lymphography of the sentinel lymph nodes***

With the continuous development of lymphography technology of SLNB in breast cancer, people can easily understand the drainage pattern of SLNs and increase the detection rate of SLNB through this way. In the bibliometric analysis, more articles have focused on lymphography combining different imaging systems with an injection of indocyanine green and confirmed this method was feasible and safe for intraoperative SLNB and could be observed in real time without long-term training (57–61). Furthermore, novel tracers and relevant imaging systems (e.g., axillary ultrasound, and computed tomography lymphography) to locate and visualize positive lymph nodes in different ways can be adopted to guide the implementation of SLNB in clinical practice (62–64).

The lymphography of the SLNs is still in the experimental stage and needed to assess and confirm by more and further clinical articles. Although this technology enhances the ability of lymph node drainage, it may complicate subsequent surgery and radiation therapy. According to the results of the research by Jung et al., the use of indocyanine green fluorescence plus radioisotope dual imaging can also improve the detection rate of SLNs after NAC (65). Consequently, the lymphography of the SLNs may not be necessary and the FNR can still be effectively reduced by using the double-tracer method of different tracers.

## 5 Limitations

Firstly, only a single database was included in the bibliometric analysis which resulted in incomplete search results. Secondly, the retrieved articles only included English language literature. Thirdly, articles from the last few years have low citation rates due to their recent publication, but this does not mean that they are not important. Despite the above limitations, the analysis of this study provides insights into current controversies and future research directions for SLNB.

## 6 Conclusion

In this study, the top 100 most cited articles on SLNB for breast cancer were analyzed through bibliometrics in combination with network visualization analysis. Notably, the US was the leading country of most cited articles, and the research hotspots focused on the quality of life and complications after surgery, the impact of neoadjuvant chemotherapy on the false-negative rate of SLNB, lymphography, and prediction of metastatic SLNs. Future research directions may include decreasing the false-negative rate and increasing the accuracy of SLNB to expand its indications through the continuous development of advanced lymphatic imaging and mapping techniques and the establishment of reasonably predictive models of lymph node metastasis.

## Data availability statement

The original contributions presented in the study are included in the article/**Supplementary Material**. Further inquiries can be directed to the corresponding authors.

## Author contributions

P-FL and PZ designed the study. KM and X-CC conducted a literature search. P-FL, PZ, KM, and X-CC analyzed data and wrote this thesis. All authors contributed to the article and approved the submitted version.

## Supplementary material

Supplementary Figure 1 Most relevant sources (top 20 journals).

Supplementary Figure 2 The top 20 publishing institutions.

Supplementary Figure 3 The three-domain diagram of authors, institutions, and countries. The size of the squares represents the number of articles published, and the linking lines to each other represent attribution.
