# Erratum: Top 100 cited classical articles in sentinel lymph nodes biopsy for breast cancer

**DOI:** 10.3389/fonc.2023.1332676

**Published:** 2023-11-14

**Authors:** Frontiers Production Office

**Affiliations:** Frontiers Media SA, Lausanne, Switzerland

**Keywords:** breast cancer, sentinel lymph node, biopsy, trends, bibliometric

Due to a production error, there was a mistake in **Figure 1** and **Figure 2** as published. **Figures 1** and 2 were swapped by mistake. The corrected order of **Figures 1** and **2** appears below. The publisher apologizes for this mistake.

**Figure 1:**
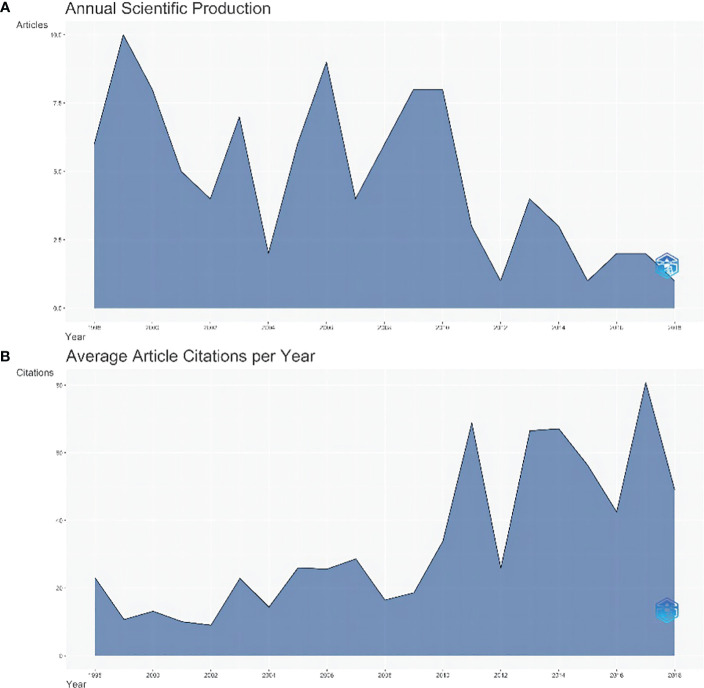
The map of annual scientific production and annual average article citations. **(A)** The annual scientific output of the top 100 most cited articles, **(B)** The average yearly number of article citations.

**Figure 2:**
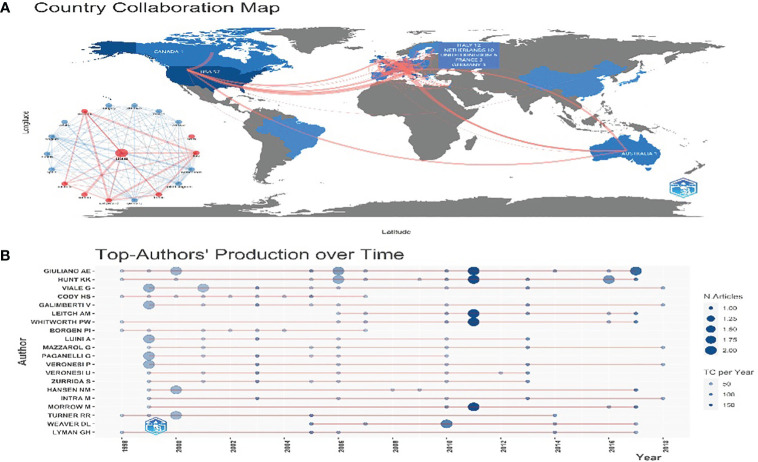
**(A)** Country Cooperation Map. The color depth represents the number of documents sent. The red line represents cooperation between countries. The thicker the line, the more the connections will be. **(B)** The top 20 authors’ production over time. The light blue circle represents the total citation (TC) per year, and the dark blue represents the number of articles.

The original version of this article has been updated.

