# Supplementary figures and images for: Top 100 cited classical articles in sentinel lymph nodes biopsy for breast cancer
Source: Front Oncol. 2023 Oct 9;13:1170464. doi: 10.3389/fonc.2023.1170464 (PMC10600391; doi:10.3389/fonc.2023.1170464)

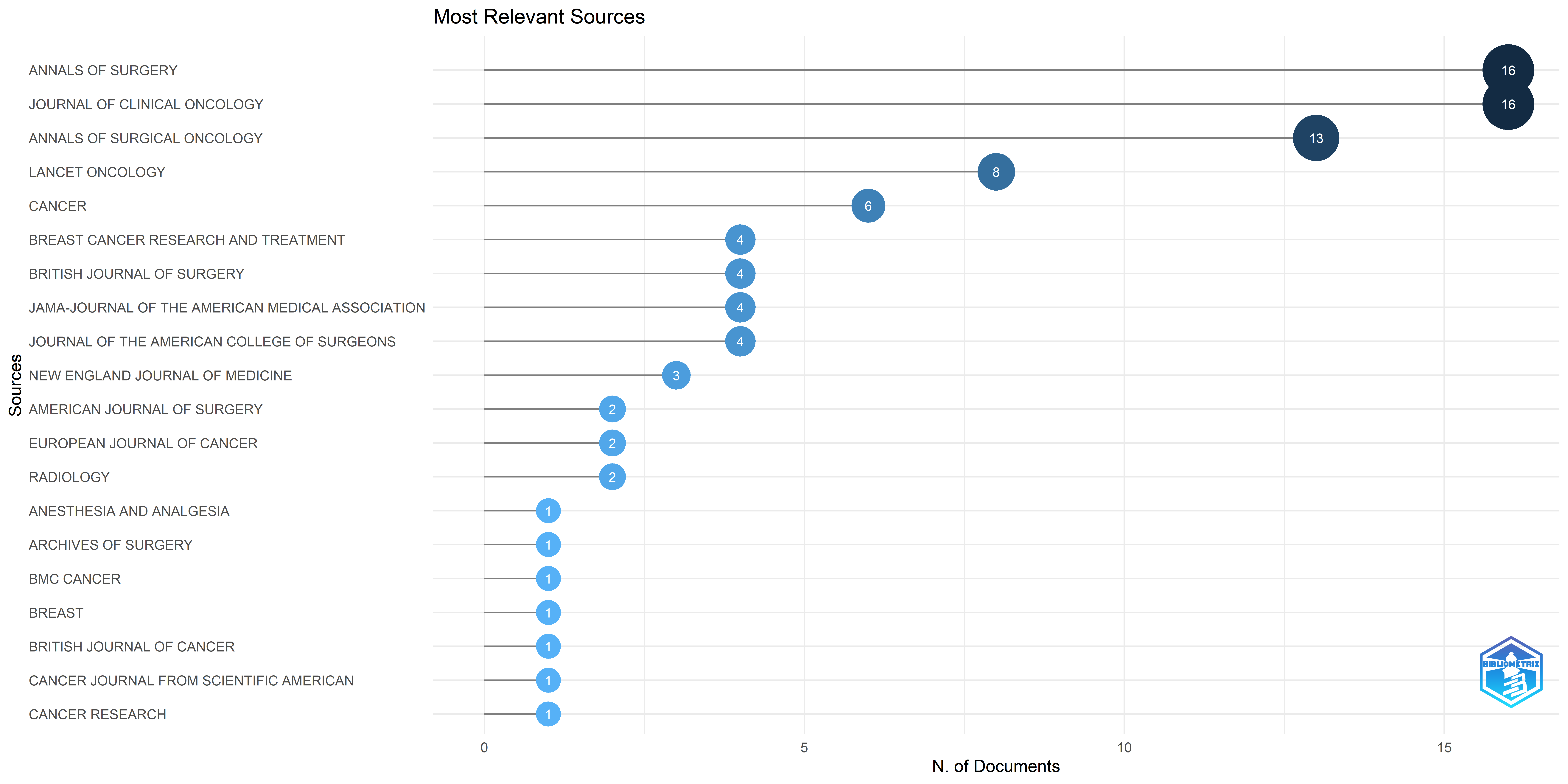

Supplement: Supplementary Figure 1 — Most relevant sources (top 20 journals). [file Image_1.png]

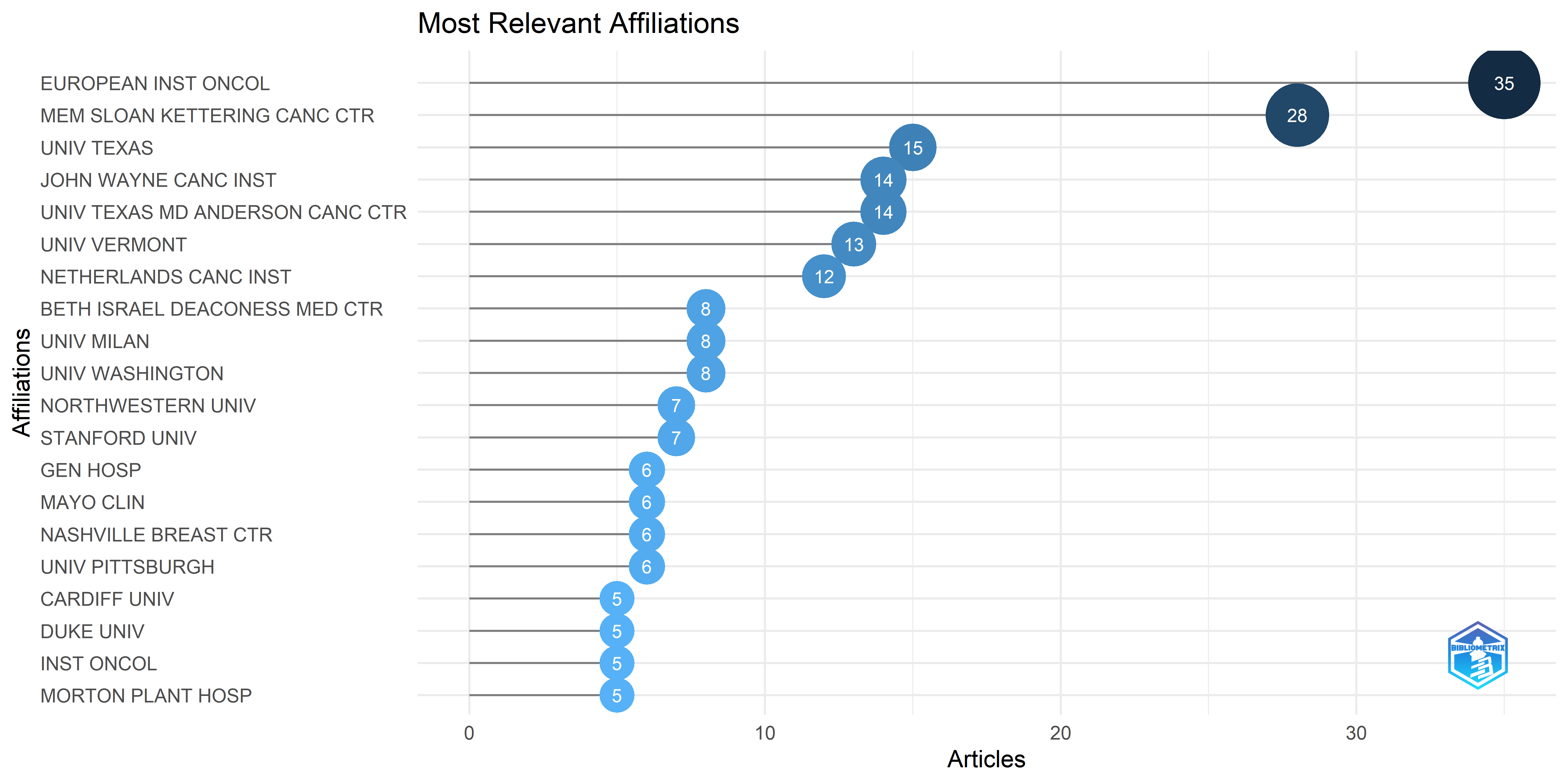

Supplement: Supplementary Figure 2 — The top 20 publishing institutions. [file Image_2.png]

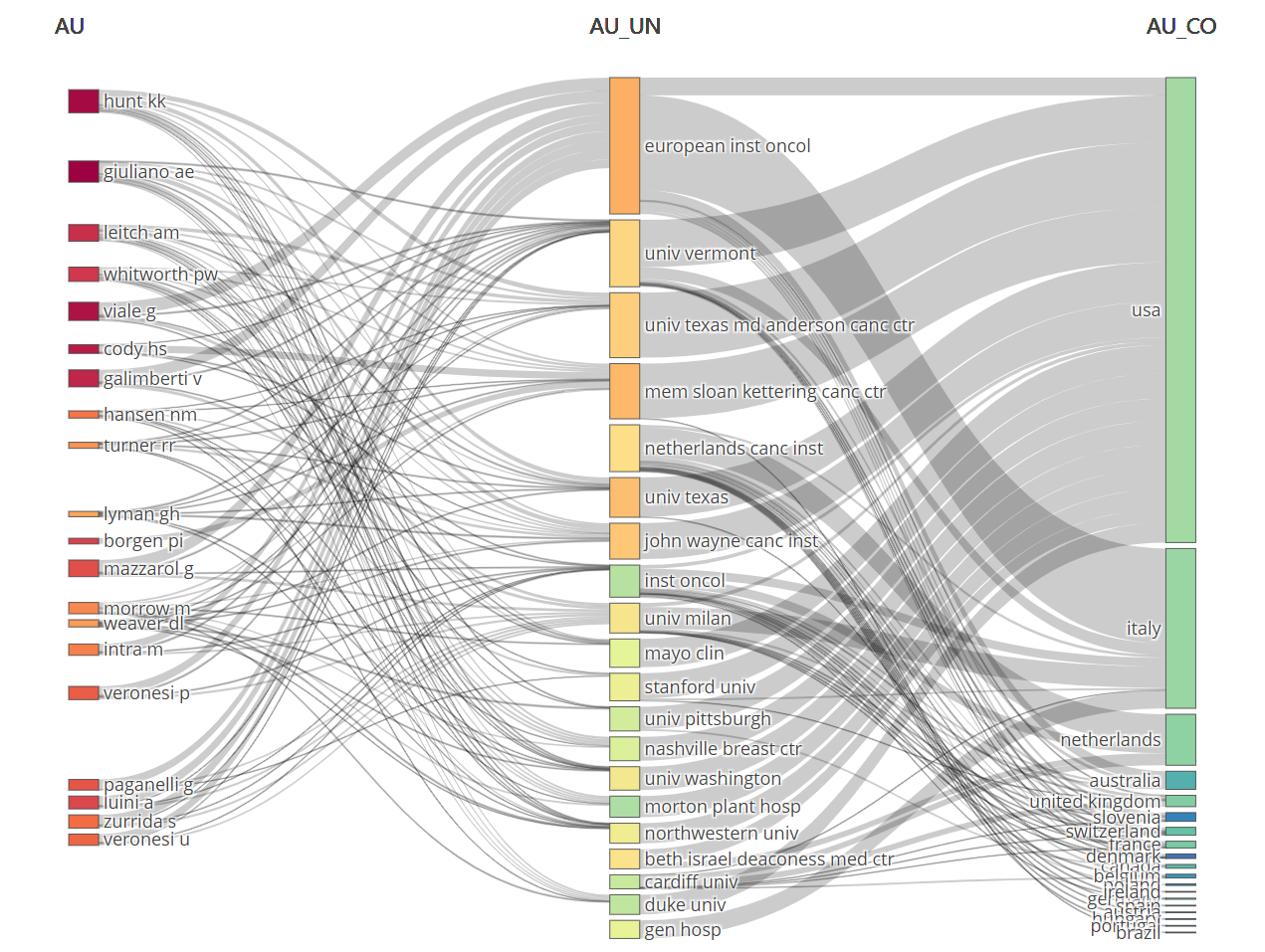

Supplement: Supplementary Figure 3 — The three-domain diagram of authors, institutions, and countries. The size of the squares represents the number of articles published, and the linking lines to each other represent attribution. [file Image_3.png]
