# Supplementary material for: Top 100 cited classical articles in sentinel lymph nodes biopsy for breast cancer
Source: Front Oncol. 2023 Oct 9;13:1170464. doi: 10.3389/fonc.2023.1170464 (PMC10600391; doi:10.3389/fonc.2023.1170464)
Supplement: Supplementary file 4 [file Table_1.doc]

**Supplementary Table 1: Calculated parameters of four clusters**

| **Cluster** | **Size** | **ISim** | **ISdev** | **ESim** | **ESdev** |
| --- | --- | --- | --- | --- | --- |
| 0 | 4 | 0.539 | 0.060 | 0.050 | 0.015 |
| 1 | 6 | 0.456 | 0.079 | 0.048 | 0.022 |
| 2 | 6 | 0.384 | 0.091 | 0.079 | 0.104 |
| 3 | 6 | 0.327 | 0.064 | 0.050 | 0.054 |

*ISim (Internal similarity); ISdev (Internal standard deviation); ESim (External similarity); ESdev (External standard deviation).*
