# Supplementary material for: Top 100 cited classical articles in sentinel lymph nodes biopsy for breast cancer
Source: Front Oncol. 2023 Oct 9;13:1170464. doi: 10.3389/fonc.2023.1170464 (PMC10600391; doi:10.3389/fonc.2023.1170464)
Supplement: Supplementary file 5 [file Table_2.doc]

| Funding Agencies | Record Count | % of 367 | County |
| --- | --- | --- | --- |
| Nih National Cancer Institute Nci | 29 | 29.000% | USA |
| United States Department Of Health Human Services | 32 | 32.000% | USA |
| National Institutes Of Health Nih Usa | 32 | 32.000% | USA |
| Nih National Institute Of Biomedical Imaging Bioengineering Nibib | 5 | 5.000% | USA |
| Nih National Institute Of Neurological Disorders Stroke Ninds | 3 | 3.000% | USA |
| American College Of Surgeons Oncology Group | 2 | 2.000% | USA |
| International Breast Cancer Study Group | 2 | 2.000% | USA |
| United States Public Health Service | 2 | 2.000% | USA |
| Amgen Inst | 1 | 1.000% | USA |
| Arbeitsgemeinschaft Fur Gynakologische Onkologie Breast | 1 | 1.000% | UK |

**Supplementary Table 2: Top 10 funding agencies.**
